# STN7 Kinase Is Essential for *Arabidopsis thaliana* Fitness under Prolonged Darkness but Not under Dark-Chilling Conditions

**DOI:** 10.3390/ijms23094531

**Published:** 2022-04-20

**Authors:** Anna Węgrzyn, Małgorzata Krysiak, Anna Kulik, Katarzyna B. Gieczewska, Radosław Mazur

**Affiliations:** 1 Department of Metabolic Regulation, Institute of Biochemistry, Faculty of Biology, University of Warsaw, Miecznikowa 1, 02-096 Warsaw, Poland; a.wegrzyn4@uw.edu.pl (A.W.); malgorzata.krysiak@uw.edu.pl (M.K.); 2 Department of Plant Anatomy and Cytology, Institute of Plant Experimental Biology and Biotechnology, Faculty of Biology, University of Warsaw, Miecznikowa 1, 02-096 Warsaw, Poland; k.gieczewska@uw.edu.pl; 3 Institute of Biochemistry and Biophysics, Polish Academy of Sciences, Pawińskiego 5a, 02-106 Warsaw, Poland; anja@ibb.waw.pl

**Keywords:** *Arabidopsis thaliana*, chilling response, darkness, dark-chilling, LHCII phosphorylation, photosynthesis, STN7 kinase

## Abstract

Reversible phosphorylation of photosystem II light harvesting complexes (LHCII) is a well-established protective mechanism enabling efficient response to changing light conditions. However, changes in LHCII phosphorylation were also observed in response to abiotic stress regardless of photoperiod. This study aimed to investigate the impact of dark-chilling on LHCII phosphorylation pattern in chilling-tolerant *Arabidopsis thaliana* and to check whether the disturbed LHCII phosphorylation process will impact the response of Arabidopsis to the dark-chilling conditions. We analyzed the pattern of LHCII phosphorylation, the organization of chlorophyll–protein complexes, and the level of chilling tolerance by combining biochemical and spectroscopy techniques under dark-chilling and dark conditions in Arabidopsis mutants with disrupted LHCII phosphorylation. Our results show that during dark-chilling, LHCII phosphorylation decreased in all examined plant lines and that no significant differences in dark-chilling response were registered in tested lines. Interestingly, after 24 h of darkness, a high increase in LHCII phosphorylation was observed, co-occurring with a significant F_V_/F_M_ parameter decrease. The highest drop of F_V_/F_M_ was detected in the *stn7-1* line–mutant, where the LHCII is not phosphorylated, due to the lack of STN7 kinase. Our results imply that STN7 kinase activity is important for mitigating the adverse effects of prolonged darkness.

## 1. Introduction

Light-dependent reactions of photosynthesis take place in a three-dimensional system of internal chloroplast membranes called the thylakoid network. Such an intricate membrane arrangement is built by the specific composition of lipids, proteins, pigments, and other organic molecules. The system of the spatial thylakoid arrangement, formed by stacked (grana) and unstacked regions, creates an organized base for biochemical and biophysical interactions between membrane components. The hierarchical organization of chlorophyll–protein (CP) complexes into supercomplexes and megacomplexes, their spatial segregation, and mutual interactions are considered the most critical factors determining the efficiency of photosynthetic light reactions and electron transfer [1]. Photosystem II (PSII) and its antenna light harvesting complex II (LHCII) are mainly found in the stacked regions, in which they form LHCII-PSII supercomplexes, composed of the dimer of the PSII core, minor light harvesting complexes (Lhcb4-6), and variable amounts of LHCII trimer (Lhcb1-3). In unstacked thylakoid regions, the monomeric photosystem I (PSI) core complex with four antennae light-harvesting complex I (LHCI) subunits (Lhca1-4) form LHCI-PSI supercomplexes. LHCI-PSI with additional Lhca proteins and multi-subunit Ndh-like chloroplast dehydrogenase (NDH) form the largest complex of thylakoid membranes. In addition, monomeric PSII without LHCII trimers and the chloroplast ATPase complex are present in unstacked stroma thylakoids. The dimeric form of the cytochromes b_6_f complex is distributed in both regions of thylakoid membranes. Thylakoid membranes also contain other proteins required for the most efficient energy distribution between photosystems and the maintenance of the redox state of chloroplasts (reviewed in, e.g., [2,3,4]).

It is known that fluctuating environmental factors such as light intensity and temperature induce rearrangements of CP complexes for maintaining the high performance of photosynthesis and redox poise inside chloroplasts (reviewed in, e.g., [5,6,7,8]). The most common mechanism behind these rearrangements is reversible phosphorylation of thylakoid proteins, which controls two important regulation mechanisms—‘state transitions’ and PSII repair cycle (reviewed in [9]).

During state transitions, the phosphorylation of LHCII is catalyzed by a membrane-bound STATE TRANSITION 7 (STN7) kinase [10]. The activity of STN7 is controlled by the redox state of plastoquinone (PQ) and cytochromes b_6_f complex, and the phosphorylation state of the kinase itself [9,11]. During enhanced excitation of PSII, the PQ pool in thylakoids becomes highly reduced and leads to activation of STN7 kinase. Phosphorylation of LHCII proteins (Lhcb1 and Lhcb2) modifies the affinity of LHCII to PSII; phosphorylated LHCII (P-LHCII) dissociates from PSII, lowering the relative size of the antenna cross section (state 2). The STN7 kinase catalytic domain is exposed to the chloroplast stroma [12], and thus LHCII phosphorylation is possible only in the stroma-exposed areas of thylakoid membranes. In the classical concept of state transitions, the P-LHCII migrates towards unstacked membranes and associates with PSI, forming a PSI–LHCI–LHCII supercomplex [13,14,15,16]. However, the novel data suggest the presence of two pools of LHCII, which are separated in stacked and unstacked membranes. Thus, the migration of P-LHCII between photosystems is limited [17,18].

LHCII proteins phosphorylation is a reversible process. Under light conditions, when the PQ pool is oxidized (e.g., preferential excitation of PSI), the activity of STN7 decreases, and constitutively active thylakoid phosphatase TAP38/PPH1 dephosphorylates LHCII components [19,20]. Dephosphorylated LHCII has a higher affinity to PSII than PSI, and as a consequence, the LHCII antenna re-associates to PSII (state 1) [19,20].

The state transitions is considered a crucial mechanism responsible for balancing the light excitation energy between the two photosystems, thus maintaining the chloroplast redox poise, especially under fluctuating light conditions. However, the state transitions or at least changes in LHCII phosphorylation were reported for various environmental stresses. For example, PEG-induced osmotic stress caused the increase in LHCII proteins phosphorylation in wheat, and this increase was higher in the drought-resistant cultivar [21]. In another study, spring wheat cultivars from the United Kingdom and Iran subjected to 14 days of water deficit showed a decrease in LHCII phosphorylation compared with the well-watered control condition [22]. Fluctuation in Lhcb2 protein phosphorylation was also observed in *Arabidopsis thaliana* subjected to salinity stress [23]. On the contrary, several reports also show the activity of STN7 kinase under dark conditions. For instance, LHCII phosphorylation and the state transitions were observed under elevated temperature (40 °C) in the dark in wheat [24] and Arabidopsis [25]. In Arabidopsis, the STN7 activation was triggered by the non-photochemical reduction of PQ by PGR5 independent antimycin A sensitive pathway [26]. LHCII phosphorylation and state transitions were also observed under dark-anaerobic conditions in Arabidopsis—in this case, the non-photochemical reduction of the PQ pool was mediated by the NDH complex [27].

Further, the low temperature was also recognized as an activator of LHCII phosphorylation in Arabidopsis [28] and runner bean [29]. In Arabidopsis, under dark-chilling conditions, an increase in the LHCII phosphorylation was transient and reached the maximum after two hours of stress. In contrast with heat stress, there were no changes in the antenna cross section of photosystems, which indicates that the migration of LHCII was somehow hampered [28].

Our previous report showed that dark-chilling treatment induces an opposite response in LHCII phosphorylation patterns in plants with different chilling tolerance [29]. In chilling-tolerant (CT) garden pea (*Pisum sativum*), we observed a decrease in LHCII phosphorylation after five days of dark-chilling, while in chilling-sensitive (CS) runner bean (*Phaseolus coccineus*), a high increase in LHCII phosphorylation was registered.

This study aimed to investigate whether different LHCII phosphorylation statuses impact the response to dark-chilling conditions in chilling-tolerant *Arabidopsis thaliana*. We used Arabidopsis mutants with disrupted LHCII phosphorylation pathways: *stn7-1*—a knockout mutant in STN7 kinase gene, *tap38-2*—a knockout mutant in TAP38/PPH1 phosphatase, and chilling-sensitive mutant *chs2-1*—a point mutation in chalcone synthase gene. In the set of mutants and wild-type Arabidopsis plants, we analyzed the pattern of LHCII phosphorylation, CP complexes’ organization, and chilling-tolerance level by a combination of molecular, biochemical, and spectroscopy techniques under dark-chilling and dark conditions. We showed that there was a decrease in LHCII phosphorylation level during dark-chilling in all examined lines, especially in the phosphorylation of Lhcb2 protein, without any significant change in chilling tolerance. Interestingly, after 24 h of darkness, a high increase in LHCII phosphorylation, especially in wt and *chs2-1* lines, was observed. This observation co-occurs with a significant F_V_/F_M_ value decrease under dark conditions in all examined lines, and the highest drop was detected for *stn7-1* mutant plants. These results imply that STN7 kinase activity and/or LHCII phosphorylation are important for mitigating the adverse effects of prolonged darkness.

## 2. Results

### 2.1. Phosphorylation Pattern of Thylakoid Proteins under Dark-Chilling and Dark Conditions

**Figure 1:**
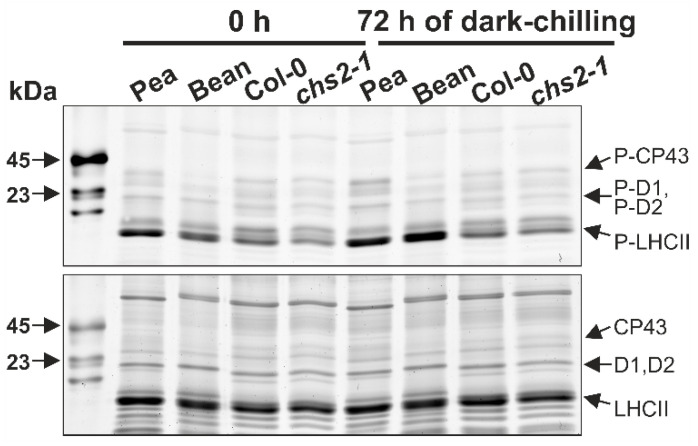
Electrophoretic analysis of thylakoid phospho-protein (**upper panel**) and protein (**lower panel**) composition in thylakoid samples isolated from chilling-tolerant pea (*P. sativum*) and Arabidopsis (*A. thaliana*) wild-type Columbia-0 (Col-0), and chilling-sensitive runner bean (*P. coccineus*) and Arabidopsis *chs2-1* plants after 72 h of dark-chilling. SDS-PAGE gels were stained with ProQ Diamond (**upper panel**) and SYPRO Ruby (**lower panel**). Analyses were carried out in a single experiment. Densitometric analysis is provided in Table S1.

In our previous research, we showed that dark-chilling induces an increase in LHCII phosphorylation in chilling-sensitive (CS) runner bean but not in chilling-tolerant (CT) pea [29]. Here, we observed a similar correlation between chilling sensitivity and LHCII phosphorylation status for CT wild-type Arabidopsis (Col-0) and CS *chs2-1* mutant (Figure 1). Compared with the level from the start of the experiment 0 h (i.e., after 16 h night period), after 72 h of dark-chilling exposure, LHCII phosphorylation increased by 21% in CS *chs2-1*, while in CT Col-0 decreased by 29%. (Figure 1). Hence, we decided to examine whether disturbances in the LHCII phosphorylation process will influence Arabidopsis chilling tolerance using phosphorylation-related mutants: *stn7-1* and *tap38-2*, lacking LHCII phosphorylation and dephosphorylation enzyme, respectively.

**Figure 2:**
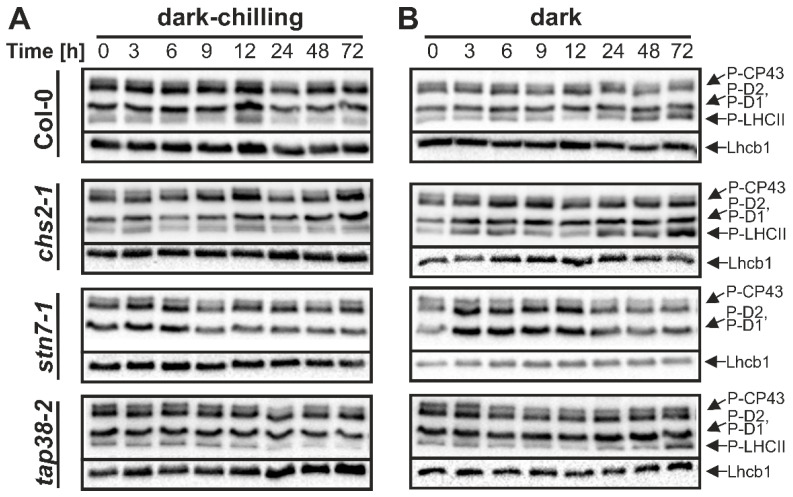
Immunodetection of thylakoid proteins phosphorylation and Lhcb1 protein level in crude thylakoid fractions isolated from wild-type Col-0, *chs2-1*, *stn7-1*, and *tap38-2* lines of Arabidopsis plants during three days of dark-chilling (**A**) and dark (**B**) treatment. SDS-PAGE was followed by immunoblotting of phosphorylated proteins with anti-phospho-threonine and Lhcb1 antibodies. Analyses were carried out in a single experiment. Densitometric analysis is provided in Table S2.

To determine how protein phosphorylation level changes during three days of dark-chilling exposure, we performed Western blot analysis using antibodies against phospho-threonine (Figure 2). We observed a slight increase in LHCII phosphorylation after 12 and 72 h of dark-chilling exposure in Col-0 and *chs2-1* lines. As expected, *stn7-1* showed no LHCII phosphorylation. For *tap38-2*, we expected to observe persistent hyperphosphorylation, regardless of the conditions [30,31]. However, a decrease in LHCII phosphorylation was observed not only in this study (Figure 2) but also before [32,33].

Interestingly, we observed an increased LHCII phosphorylation also under dark conditions at optimal temperature (Figure 2B). Col-0 showed 2.2 times more intense LHCII phosphorylation after 72 h of dark, compared with 0 h. In *chs2-1*, LHCII phosphorylation fluctuated under dark conditions but was always at least 2 times higher than at the start of the experiment, and after 72 h of darkness, LHCII phosphorylation in *chs2-1* was even 6.2 times higher (Figure 2). In *tap38-2*, LHCII phosphorylation slightly decreased during the first 12 h of darkness and then suddenly increased, reaching over 2-fold enhancement after 72 h compared with 0 h (Figure 2).

**Figure 3:**
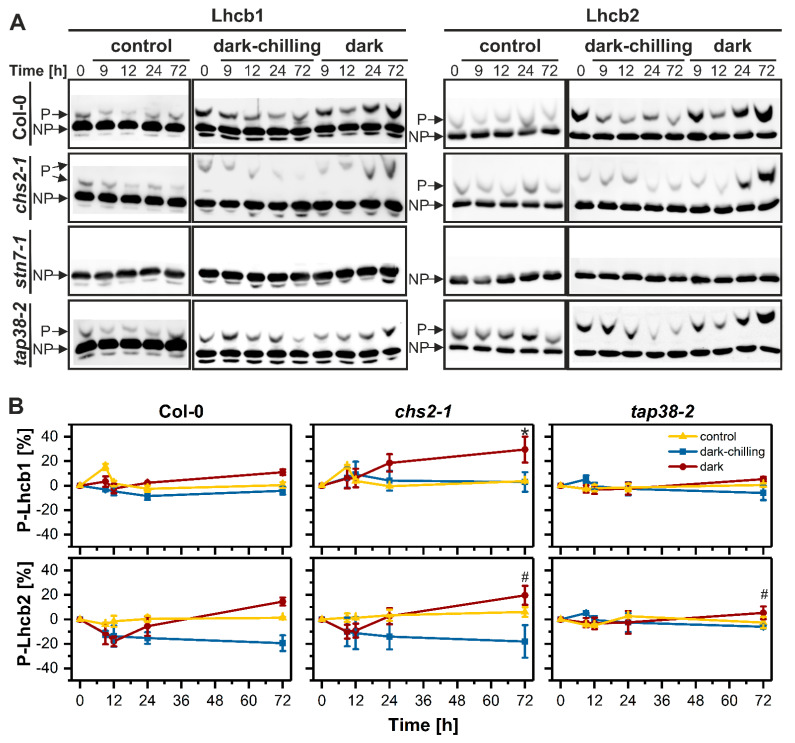
Immunodetection analysis of Lhcb1 and Lhcb2 proteins phosphorylation level in crude thylakoid fractions isolated from wild-type Col-0, *chs2-1*, *stn7-1*, and *tap38-2* lines of Arabidopsis plants from control, dark-chilling, and dark conditions. (**A**) Representative blots presenting separation of phosphorylated (P) and non-phosphorylated (NP) forms of Lhcb1 and Lhcb2 proteins produced by Phos-Tag™ PAGE electrophoresis. (**B**) Changes in Lhcb1 and Lhcb2 phosphorylation level calculated as P/(P + NP) and normalized to 0% at time point 0 h. Data are mean values ± SE from three (dark-chilling and dark) and two (control) independent experiments. Results marked with an asterisk and hash differ significantly at *p* = 0.05 (one-way ANOVA with post hoc Tukey test) from time point 0 h or between dark-chilling and dark conditions, respectively.

In Col-0, *chs2-1* and *tap38-2* LHCII phosphorylation fluctuated under dark and dark-chilling conditions. However, we also observed some fluctuations in Lhcb1 protein content in all tested lines, which influence the interpretation of phosphorylation changes. For this reason, we used the PhosTag^TM^ PAGE approach, since PhosTag^TM^ added to the separating gel slows down the migration of phosphorylated proteins, which results in two bands visible after Western blot—an upper one corresponding to phosphoprotein and a lower one to unphosphorylated protein. Thus, this method gives the absolute content of the phosphoprotein of interest and is insensitive to unequal protein loading. Based on data presented in Figure 2, we selected time points where changes in LHCII phosphorylation were the most significant (9 h, 12 h, 24 h, 72 h) and used them for further detailed analysis of the phosphorylation level of two major LHCII proteins: Lhcb1 and Lhcb2 (Figure 3). Under control conditions, we observed the increase in Lhcb1 phosphorylation after 9 h, with a subsequent decrease to the base level in Col-0 and *chs2-1* lines. In contrast, phosphorylation of Lhcb2 was stable during three days in all examined lines (Figure 3B). All time points from control conditions were collected at the end of the dark phase of the day/night period, and the lack of increased Lhcb proteins phosphorylation was expected. The transient increase in phosphorylation level of Lhcb1 but not Lhcb2 protein after 9 h can be explained by the higher affinity of TAP38/PPH1 phosphatase to P-Lhcb2. Dark-chilling did not induce significant changes in Lhcb1 nor did it induce Lhcb2 phosphorylation in any examined lines. Only a slight and transient increase in P-Lhcb1 after 9 h of dark-chilling in *chs2-1* and *tap38-2* mutants was observed, while the P-Lhcb2 content decreased during dark-chilling in wt and *chs2-1* lines (Figure 3B). On the contrary, for both wt and *chs2-1* lines, prolonged darkness induced a constant increase in P-Lhcb1 content, reaching 10% and 20% of maximal phosphorylation after 72 h, respectively. The Lhcb2 phosphorylation pattern under dark conditions was more complex; the P-Lhcb2 level first decreased, then from 12 h of dark exposure started to increase, reaching 15% and 20% of maximal phosphorylation in Col-0 and *chs2-1*, respectively (Figure 3B). In *tap38-2*, prolonged dark exposure induced an identical pattern of Lhcb1 and Lhcb2 phosphorylation changes as in other lines, but with a lower magnitude—around 5% phosphorylation increase after 72 h compared with time 0 h (Figure 3B).

### 2.2. Organization of the Chlorophyll–Protein (CP) Complexes under Dark-Chilling and Dark Conditions

**Figure 4:**
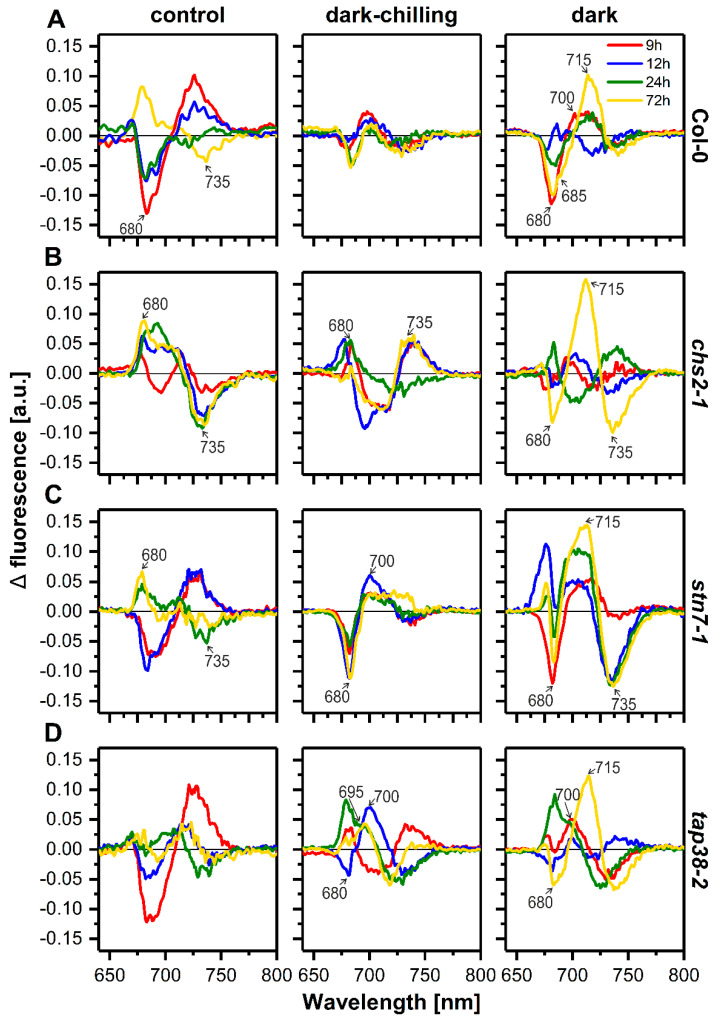
Analysis of low-temperature fluorescence spectra of crude thylakoid fractions isolated from wild-type Col-0, *chs2-1*, *stn7-1*, and *tap38-2* lines of Arabidopsis plants from control, dark-chilling, and dark conditions. Comparison of the normalized fluorescence emission difference spectra (time point minus time 0 h) for the Col-0 (**A**), *chs2-1* (**B**), *stn7-1* (**C**), *tap38-2* (**D**) lines. Presented spectra are the mean values from two independent experiments.

Analysis of CP complexes organization was performed using low temperature (77 K) fluorescence emission spectroscopy. Thylakoids’ typical fluorescence emission spectrum is composed of two major bands centered at around 685 and 730 nm, corresponding to chlorophylls (Chl) bound to PSII and PSI, respectively (Figure S1). We identified six main components of the spectra: the trimeric and monomeric LHCII antennae (680 nm), the core complex of PSII (685 nm), the core antenna complex of PSII (695 nm), the aggregated trimers of LHCII (700 nm), PSI reaction center complex (715–720 nm), and LHCI antennae (735 nm) (Figure S1) [34]. Based on recorded fluorescence spectra, we (i) calculated arithmetic difference spectra ‘time point minus 0 h’ and (ii) performed mathematical deconvolution with Gaussian components for all lines and experimental conditions (Figure 4 and Figure S2).

Under control conditions in all examined lines, we observed similar changes in the difference spectra, i.e., the decrease in 680 nm and the increase in 735 nm bands (Figure 4 and Figure S2). However, the *chs2-1* plants exhibited a distinct pattern of difference emission spectra after 9 and 12 h, indicating different CP complexes organization after day period compared with other lines (Figure 4, left panels). No significant differences in CP complexes organization were observed under dark-chilling conditions in Col-0 (Figure 4A, middle panel). Contrarily, dark exposure induced a decrease in fluorescence at 680 nm (9 h, 72 h), 685 nm (24 h, 72 h), and 735 nm (72 h) with a simultaneous increase in fluorescence at 700 nm (9 h) and 715 nm (72 h) (Figure 4A, right panel). It shows that prolonged darkness in Col-0 caused the reduction in the PSII antenna size via LHCII aggregation (in a short time) and an enlargement in the PSI core emission (after a long time).

In *chs2-1* plants, after 9 h of dark-chilling, the fluorescence signal increase at 680 nm and 735 nm and a decrease at around 705 nm were observed, and these changes were mostly stable during dark-chilling (Figure 4B, middle panel). Under prolonged dark conditions in *chs2-1* thylakoids, we did not observe significant CP complexes remodeling during the first 24 h. In comparison, after 72 h of the dark, a high increase at 715 nm, along with a simultaneous decrease at 680 nm and 735 nm, was found. (Figure 4B, right panel). In *stn7-1* plants, only a high reduction in fluorescence at 680 nm and an increase at 700 nm, with no effect on PSII and PSI core complexes, were observed during dark-chilling (Figure 4C, middle panel). During prolonged darkness, more dynamic remodeling of the CP complexes was observed. After 9 h of the dark, the fluorescence decrease at 680 nm and the increase at 715 nm were observed. This effect lasted for 72 h of dark, with a constant increase at 715 nm and a decrease at 735 nm (Figure 4C, right panel). In *tap38-2* plants, an opposite effect of dark-chilling was observed compared with *stn7-1*. Fluorescence at 680 nm (9 h, 24 h), 695 nm (72 h), and 700 nm (12 h) increased, which corresponds to the enrichment of PSII–LHCII supercomplexes, while changes in LHCI-related fluorescence were oscillating below 5% (Figure 4D, middle panel). During the prolonged dark, almost identical CP remodeling was observed as in dark-chilling, with two differences: fluorescence at 700 nm (LHCII aggregation) happened earlier, and a high increase in fluorescence at 715 nm (PSI core) occurred after 72 h (Figure 4D, right panel). What is worth mentioning is that we observed almost identical changes in fluorescence spectra in all tested lines after 72 h of darkness: the decrease in LHCII (680 nm) and LHCI (735 nm) with a simultaneous increase in PSI core (715 nm) (Figure 4, right panels).

Additional analysis of low-temperature fluorescence spectra by Gaussian deconvolution (Figure S2) showed almost identical changes in the contribution of five main components of the emission spectra, as described above. We confirmed that the most pronounced changes in CP complexes organization were observed under dark conditions and consisted of (i) decrease in PSII-associated bands (F685, F695) (Figure S2A,B), (ii) increase in PSI core (F720) (Figure S2D), and (iii) increase in aggregated LHCII (F700) in *stn7-1* line (Figure S2C).

### 2.3. Lipid Peroxidation and Photosynthetic Pigments Content under Dark-Chilling and Dark Conditions

**Figure 5:**
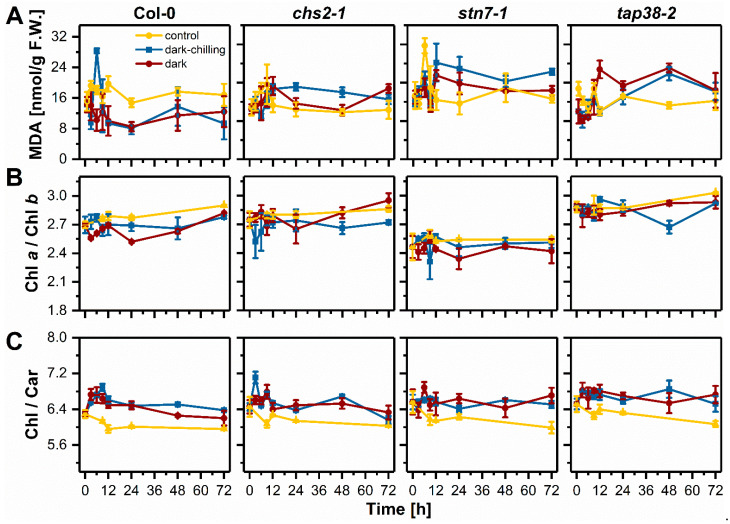
Analysis of lipid peroxidation and photosynthetic pigment ratios in leaves of wild-type Col-0, *chs2-1*, *stn7-1*, and *tap38-2* lines of Arabidopsis plants from control, dark-chilling, and dark conditions during three days of the experiment. (**A**) Changes in thiobarbituric acid-reactive substances (TBARS) measured in terms of malondialdehyde (MDA) content; (**B**) chlorophyll (Chl) *a* to chlorophyll *b* ratio; (**C**) total chlorophyll to carotenoids (Car) ratio. Data are mean values ± SE from three (dark-chilling and dark) or two (control) independent experiments. Significant differences in one-way ANOVA with post hoc Tukey test at *p* = 0.05 were not reported. F.W., fresh weight.

Malondialdehyde (MDA) is the end product of lipid peroxidation, and its content is directly related to the level of membrane damage [35]. To estimate the influence of dark-chilling and dark conditions on the membrane status, we measured the extent of lipid peroxidation in terms of MDA level (Figure 5A). Under control conditions, the MDA level was relatively stable in all examined lines except for *stn7-1*, where after 6 h of light, there was a high increase in MDA, which came back to base level after 12 h (8 h of light followed by 4 h of dark) (Figure 5A). Under dark-chilling conditions after 6 h, there was an increase in MDA content in Col-0, which was quickly relaxed—after 12 h of dark-chilling, the MDA level was similar to the level at time 0 h (Figure 5A). In mutants, the MDA level was increasing during the first 12 h, and after 72 h of dark-chilling, it was at least 100% higher compared with Col-0 (Figure 5A). Prolonged dark did not change the MDA level in Col-0, while in mutants, MDA changes in the function of time were similar to those observed for dark-chilling conditions (Figure 5A).

Analysis of Chl *a* to Chl *b* (Figure 5B) as well as total Chl to carotenoids (Car) ratios (Figure 5C) showed no significant changes during experimental conditions in all examined lines, which indicate a lack of CP complexes degradation. Lower or higher Chl *a*/Chl *b* ratios compared with Col-0 were observed for *stn7-1* and *tap38-2* plants, respectively. These differences were previously reported and are characteristic of these mutants [36].

### 2.4. Photochemical Efficiency under Dark-Chilling and Dark Conditions

**Figure 6:**
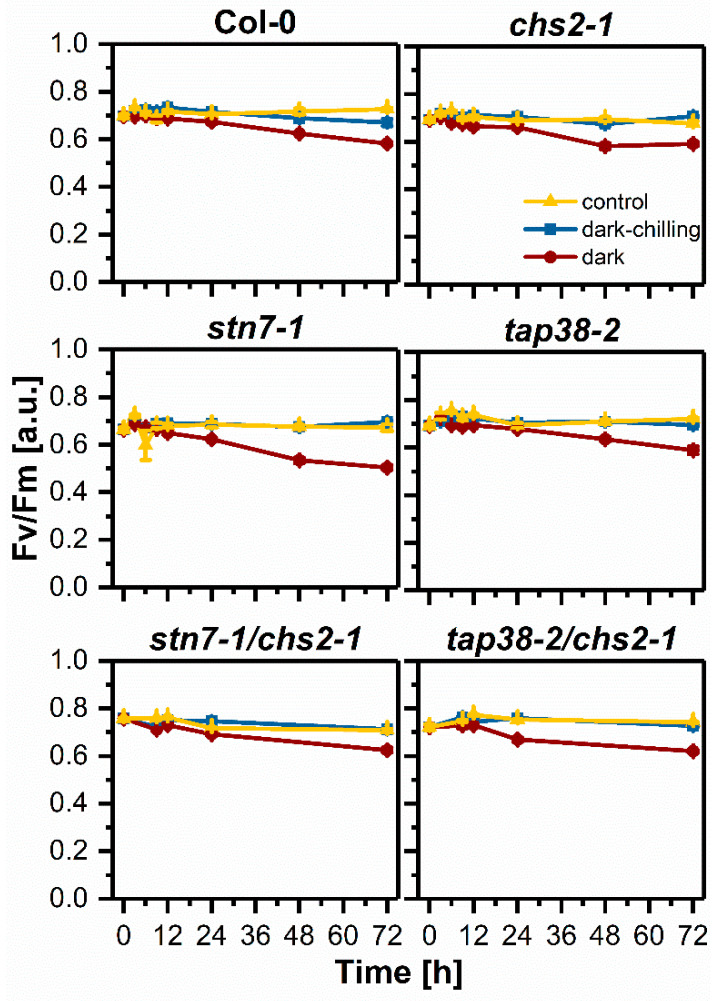
Analysis of chlorophyll *a* fluorescence in vivo in wild-type Col-0, *chs2-1*, *stn7-1*, *tap38-2*, *stn7/chs2-1*, and *tap38/chs2-1* lines of Arabidopsis plants from control, dark-chilling, and dark conditions during three days of the experiment. The F_V_/F_M_ parameter was measured in leaves adapted to darkness. Data are mean values ± SE from three (dark-chilling and dark) or two (control) independent experiments. Results of statistical analysis (one-way ANOVA with post hoc Tukey test at *p* = 0.05) are shown in Figure S3.

Analysis of photochemical parameters obtained from in vivo Chl *a* fluorescence is one of the easiest ways to determine the adverse effects of various environmental stresses on photosynthetic apparatus. We measured the F_V_/F_M_ parameter under dark, dark-chilling, and control conditions (Figure 6). As expected, a regular short-day photoperiod caused no change in the F_V_/F_M_ value in Col-0 and all mutants, except *stn7-1* after 6 h, where the F_V_/F_M_ decrease appeared, which correlated with increased MDA level (Figure 5A). There was no decrease in F_V_/F_M_ values under dark-chilling conditions, except for Col-0 plants after 72 h. The high F_V_/F_M_ decrease was detected under prolonged dark conditions. The negative effect of darkness on F_V_/F_M_ was visible after 12 h and accumulated in 24 and 72 h in all tested lines, with the highest decrease in the *stn7-1* mutant. To check the combined effect of chilling sensitivity and disorders in LHCII phosphorylation on photosynthetic performance, we created the double mutants *stn7-1/chs2-1* and *tap38-2/chs2-1*. Obtained plant lines possessed the phenotype of a single *chs2-1* mutant—i.e., retarded growth. In both double mutants, similar to single ones, dark-chilling did not cause any significant changes in F_V_/F_M_ values. Under dark conditions, the decrease in F_V_/F_M_ in *stn7-1/chs2-1* and *tap38-2/chs2-1* was similar to that observed in *chs2-1* and *tap38-2* but not *stn7-1* (Figure 6).

**Figure 7:**
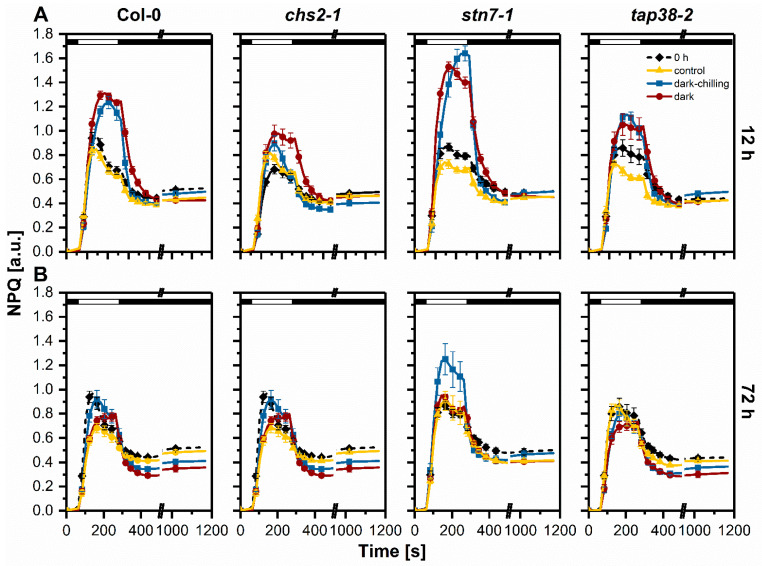
Analysis of chlorophyll *a* fluorescence in vivo in wild-type Col-0, *chs2-1*, *stn7-1*, and *tap38-2* lines of Arabidopsis plants from control, dark-chilling, and dark conditions. The non-photochemical quenching parameter (NPQ) was calculated from measurements after 12 (**A**) and 72 h (**B**) of control, dark-chilling, and dark treatment. Data are mean values ± SE from three (dark-chilling and dark) or two (control) independent experiments. Black and white bars on plots represent dark and illumination phases of the measurement, respectively. For a clear presentation of plots, one out of every two consecutive points was omitted. Results of statistical analysis (one-way ANOVA with post hoc Tukey test at *p* = 0.05) are shown in Figure S4.

It is known that various environmental stresses upregulate non-photochemical processes in the photosynthesis light-dependent phase. Thus, we analyzed the changes in the non-photochemical quenching parameter (NPQ) at two time points, 12 and 72 h (Figure 7). Under steady-state light conditions (i.e., at the end of actinic illumination, indicated as white bars in Figure 7), the NPQ value for Col-0 was 50% higher after 12 h of dark-chilling and dark exposure compared with control conditions and also to the beginning of the experiment (Figure 7A). The rise in NPQ was faster for dark conditions, and the NPQ value remained almost stable towards the end of illumination (Figure 7). However, after 72 h of dark-chilling or dark, NPQ values were similar to those from 0 h (Figure 7B). The rise in NPQ was faster for dark-chilling conditions, but for prolonged dark, the increase was more gradual and lasted throughout the illumination (Figure 7B). After the dark recovery phase, the NPQ values for 12 h and 72 h of dark and dark-chilling conditions were lower compared with time 0 h (Figure 7A,B).

Over 50% increase in NPQ with similar rising kinetics was also observed in the *stn7-1* mutant after 12 h of dark-chilling and dark conditions (Figure 7A). After 72 h of dark-chilling, the NPQ decreased but still was higher than in 0 h, while after 72 h in the dark, the NPQ was similar to the control values (Figure 7B). NPQ kinetics in *chs2-1* plants did not differ from control conditions in both analyzed time points and experimental conditions (Figure 7A,B). The *tap38-2* plants after 12 h exposition to dark-chilling or dark conditions presented an increase in NPQ similar to Col-0 plants, but with lower magnitude (Figure 7A), and after 72 h, there was no differenced compared with control (Figure 7B).

Analysis of qE—the high energy component of NPQ [37]—showed an increase in qE during the first 12 h of dark-chilling in all tested lines, *stn7-1* in particular. After 12 h, the slow decrease or stabilization of qE was observed (Figure S5, top panels). Under prolonged dark, the qE also increased in all tested lines, but with a 6 h delay compared with dark-chilling. (Figure S5, top panels). The qI—the photoinhibition component of NPQ [37]—presented relatively stable values, with a downward trend over time, especially under dark conditions, in all tested plants (Figure S5, bottom panels).

## 3. Discussion

Low temperature is one of the abiotic stress factors that affects plant growth and productivity. Tolerance of plants to low temperature varies between species and cultivars depending on their evolutionary background. According to the temperature range, plants have been divided into chilling-sensitive (CS) plants, susceptible to temperatures below 12 °C, and chilling-tolerant (CT) plants, resistant to low but non-freezing temperatures. Chloroplasts are the primary target of chilling, which causes the decrease in photochemistry and Calvin–Benson–Bassham cycle enzymes activity and the increase in ROS level and, consequently, the imbalance of chloroplast redox poise. What should be emphasized is that chilling in the light and the dark reveals substantial differences both in the scale of inhibition of photosynthesis and the primary mechanisms involved [38,39,40]. Light-chilling is a type of photoinhibition in which observed changes are a mixture of chilling and light stress exposure. In contrast, changes observed under dark-chilling conditions are induced only by chilling stress.

We showed here that in CT Arabidopsis Col-0 plants under dark-chilling conditions, the LHCII phosphorylation level decreases (Figure 3), and such an effect was previously reported for Arabidopsis [28,41] and CT pea after five [29] or three (Figure 1) days of dark-chilling. A decrease in LHCII phosphorylation during dark-chilling was also observed in *tap38-2* and CS *chs2-1* Arabidopsis mutants (Figure 3). In contrast, the opposite effect was reported for CS runner bean after different dark-chilling periods (Figure 1) [29]. Lack of the LHCII phosphorylation increase under dark-chilling stays in agreement with previously reported data showing that in the dark STN7 kinase is inactive [11,42]. Moreover, the stress level induced upon dark-chilling conditions might not disturb the redox equilibrium enough to activate the non-photochemical PQ reduction necessary for STN7 activation. It is also possible that an increase in LHCII phosphorylation occurs during dark-chilling, but in CT plants, it is a temporary phenomenon [28], and after 9 h of dark-chilling, such effect is no longer observed [41]. The other possible explanation for the lack of LHCII phosphorylation might be restricted access to the STN7 substrate related to membrane fluidity decrease and/or structural reorganization of thylakoid membranes leading to the area reduction in the stroma-exposed membranes. However, this hypothesis needs further experimental evidence. The lack of expected increase in LHCII phosphorylation in CS *chs2-1* Arabidopsis mutant might be explained by the type of chilling stress applied in this study. The *chs2-1* mutant has a point mutation in the chalcone synthase gene—an essential enzyme in anthocyanin’s biosynthetic pathway. Anthocyanin’s protective role under light-chilling conditions is well described [35,43,44,45]. One of its functions under stress conditions is protection of photosynthetic apparatus against over-reduction by limiting the amount of light reaching photosystems through partial reflection and absorption of light quanta [46]. Thus, we postulate that under dark-chilling conditions, the stress level was too low to reveal the chilling-sensitive phenotype of the *chs2-1* mutant.

Analysis of response to the dark-chilling in Col-0, *stn7-1*, and *tap38-2* lines did not show significant differences between plant lines. The F_V_/F_M_ values remained stable during 72 h of dark-chilling; only a slight decrease in Col-0 after 3 days was detected (Figure 6). In *stn7-1/chs2-1* and *tap38-2/chs2-1* double mutants, the F_V_/F_M_ values also remained stable during 72 h of dark-chilling (Figure 6). Biochemical analysis showed that the Chl *a*/Chl *b* ratio (Figure 5B) and lipid peroxidation (Figure 5A) after 72 h of dark-chilling had similar values as at the beginning of the experiment. In addition, there was no evidence of direct fluorescence emission from Chl *b* molecules (signal around 660 nm) in the fluorescence spectra of thylakoid samples (Figure 4). All these data indicate that CP complexes are not degraded under applied experimental conditions. However, analysis of low-temperature fluorescence spectra showed that under the dark-chilling, reorganization of CP complexes takes place (Figure 4 and Figure S2). The difference spectra of Col-0 and *chs2-1* from dark-chilling conditions had the same features as reported previously for CT pea and CS runner bean, respectively [29]. This indicates that CP complexes’ reorganization during dark-chilling proceeds in the same direction in species belonging to the same chilling-tolerant group [47,48].

Under low-temperature conditions, the membrane fluidity decreases, which lowers the diffusion speed inside the thylakoid membrane, and the reorganization of CP complexes is less pronounced. It was shown the membrane fluidity modulates the PQ pool oxidation rate [49] and thus can influence the activation of STN7. Moreover, it was revealed that phosphorylation increases protein mobility inside the thylakoid membrane [50]. Thus, its low level promotes aggregation of the LHCII (700 nm band in fluorescence difference spectra) (Figure 4). This effect is visible in dark-chilled Col-0 and *stn7-1* plants (Figure 4A,C and Figure S2). The most complex changes in 77K spectra were observed in *tap38-2* plants (Figure 4D and Figure S2), probably due to the higher base level of phosphorylated LHCII (Figure 3A), and thus different supramolecular organization of CP complexes.

In contrast with dark-chilling conditions in Col-0 and *chs2-1* plants, we observed an increase in LHCII phosphorylation level after 72 h in darkness at optimal temperature (Figure 3). As mentioned above, LHCII phosphorylation in the dark needs a reduced form of PQ, which can be synthesized without the photochemical activity of the photosystems [26,51]. Light-independent PQ reduction can be achieved by the activity of NADPH:plastoquinone oxidoreductase (NDH) and ferredoxin:plastoquinone oxidoreductase (FQR), or by direct reduction where ascorbate is as an electron donor [52]. Both mentioned enzymes need NADPH (directly or indirectly), which can be supplied by starch breakdown and the activity of the oxidative pentose phosphate pathway [53,54]. It is known that the carbohydrate metabolism depends on the daily cycle. Starch synthesis maintains a linear increase during the day, while at night, it steadily decreases to 5% of the initial content at the end of the dark period [55]. When the process of photosynthetic CO_2_ assimilation remains inactive during prolonged darkness, the starch reserve is depleted. In consequence, reduction in chloroplast stroma and photosynthetic electron transport chain may occur [56]. This reduction modulates the reduction status of thioredoxins and NADPH-dependent thioredoxin reductase C (NTRC), which control the redox metabolism inside chloroplasts [6]. Reduced NTRC can activate the NDH [57], which may lead to PQ reduction and activation of STN7 kinase.

Reorganization of CP complexes after 72 h of dark conditions is more evident than under dark-chilling (Figure 4 and Figure S2). We observed an increase in the PSI core-associated band linked with a decrease in 735 nm ones in all tested plant lines. PSII-associated fluorescence bands showed, except for the *stn7-1* line, some fluctuations (positive and negative bands in successive time points), but after 72 h, there was a negative band at 680 nm in all lines (Figure 4 and Figure S2). It indicates that reduction in the PSII antenna size via LHCII aggregation and enlargement of the PSI core are the effects of prolonged darkness in the wild-type Arabidopsis plants. What is worth noting is that in *stn7-1* plants, the shapes of the difference spectra are similar in all measured time points (9, 12, 24, and 72 h). It means that *stn7-1*’s response to the dark conditions is much faster than other tested lines. This correlates with F_V_/F_M_ values, which decreased much faster in the *stn7-1* line compared with other plants (Figure 6). Moreover, the F_V_/F_M_ value in *stn7-1* mutant after 72 h of the dark is much lower (about 16%) compared with other lines (Figure 6). Our additional analysis of the qL parameter showed that the fraction of the open PSII reaction centers is quite stable during dark conditions (Figure S6). On the contrary, under dark conditions, the F_0_/F_M_ ratio rises (Figure S7), suggesting the increasing population of PSII antenna not energetically coupled to the reaction centers [58].

Despite the similar CP complexes reorganizations observed after 72 h of dark treatment, the F_V_/F_M_ was considerably lower in *stn7-1* plants compared with other lines. This observation challenges the hypothesis that the phosphorylated form of LHCII is needed to sustain the supramolecular organization of photosynthetic apparatus under prolonged dark conditions. We postulate that STN7 kinase activity is one of the possible routes for utilization of reduced PQ accumulated in thylakoid membranes due to the activity of alternative electron transport pathways. Lack of STN7 kinase activity in *stn7-1* plants leads to over-reduction in thylakoid membranes [59] and initiation of ROS accumulation [59], in consequence, compromising the capacity of photosynthetic light reactions (Figure 6, Figure 7, and Figure S5). However, the link between STN7 kinase activity and plant fitness under prolonged dark might be more complex. Besides Lhcb1 and Lhcb2, other substrates for STN7 kinase have been also identified. It was shown that STN7 phosphorylates the Lhcb4 (CP29 minor antenna of PSII) [60], ferredoxin:NADP^+^ oxidoreductase (FNR), 30S ribosomal protein S7 (Rps7), and ATP-dependent Clp protease proteolytic subunit 3 (CLPP3) [61]. STN7 is also recognized, together with STN8 kinase, as kinase phosphorylating large Rubisco subunit, FtsH11 protease, and RNA-binding protein RNP29 [61]. These data indicate that maintaining the excitation balance between photosystems is not the only function of STN7 and that its role in other metabolic and signaling pathways in chloroplasts should be considered.

Summarizing, the results presented in this report indicate that STN7 kinase activity is important for mitigating the adverse effects of prolonged dark conditions.

## 4. Materials and Methods

### 4.1. Plant Lines, Growing Conditions, and Dark-Chilling Treatment

Seeds of *Arabidopsis thaliana* mutants *chs2-1* (CS6298) [62], *stn7-1* (SALK_073254) [10], and *tap38-2* (SALK_025713) [20] were obtained from The Eurasian Arabidopsis Stock Center and checked for homozygosity. The double mutants *stn7-1/chs2-1* and *tap38-2/chs2-1* were obtained by crossing single mutants lines, and seeds from the F3 generation were used for analysis. Primers used for genotyping of Arabidopsis mutants: LPSTN7-1–GAGCTTGTGGGAATAGCTGTG; RPSTN7-1–TAGTTGAACATGCGTGAGTCG; LPTAP38-2–TCGGCTTGTGCATATGAATTC; RPTAP38-2–TTGCATGAGAAGCAACACAAG, CHS2-1F–GATTGACCTTGTATATGAGGTGG, CHS2-1R–CACTCATCTTTGTCCCTTCCTTTTGAA.

Plants were grown in peat pellets for 6 weeks in an 8 h photoperiod at 22 °C/18 °C (day/night) at PAR of 80 µmol photons m^–2^ s^–1^. For dark-chilling treatment, the detached rosettes were placed in Dewar flasks (4 °C, 100% relative humidity) for three days. For dark and control conditions, plants were kept in darkness at 22 °C or in a growing chamber under photoperiod conditions for three days, respectively. Samples were collected at selected time points; the time zero was collected after the night period just before the light was on.

### 4.2. Preparation of Thylakoid Membranes

Thylakoid membranes were isolated by homogenization in a buffered isotonic medium and subsequent centrifugation as described previously [63]. The chlorophyll concentration was quantified spectrophotometrically after extraction with 80% (*v*/*v*) acetone [64].

For isolation of crude thylakoid fraction, harvested leaves were frozen in liquid nitrogen and ground to a fine powder in the homogenization buffer (20 mM Tricine-NaOH pH 7.5, 330 mM sorbitol, 15 mM NaCl, 4 mM MgCl_2_). Homogenates were filtered through nylon filters (50 µm) and centrifuged for 2 min (200× *g*, 4 °C). The supernatant was centrifuged again for 5 min (7000× *g*, 4 °C). The pellet was suspended in a small amount of homogenization buffer. The concentration of chlorophyll was quantified as described above.

### 4.3. Electrophoretic Techniques

Thylakoid samples containing 1 µg of Chl after denaturation in Laemmli buffer were loaded onto polyacrylamide gels and separated by the standard SDS-PAGE electrophoresis protocol. Separated proteins were transferred to polyvinylidene fluoride (PVDF) membrane followed by blocking and incubation with primary antibodies against Lhcb1 and Lhcb2 proteins (both from Agrisera, Vännäs, Sweden, Catalog Number AS01 004 and AS01 003, respectively) and phospho-threonine residue (Cell Signaling Technology, Danvers, MA, USA, Catalog Number #9386). Visualization was obtained using anti-rabbit HRP-conjugated secondary antibody (Agrisera Catalog Number AS09 602) and ECL Detection System. In-gel phospho-protein and protein staining were performed using the ProQ^®^-Diamond and SYPRO^®^ Ruby, respectively, according to the manufacturer’s (Invitrogen™, cat. no MPM33305) protocol.

### 4.4. Two-Layer Phos-Tag^TM^ PAGE

Two-layer Phos-Tag electrophoresis was performed according to the modified method described in [14]. The gels were prepared as follows: (i) heavy gel solution: 357 mM Bis-Tris (pH 6.8), 30% (*w*/*v*) glycerol, 7% (*w*/*v*) acrylamide/bis-acrylamide (37.5:1), 0.05% (*v*/*v*) N,N,N’,N’-tetramethylethylenediamine (TEMED), 0.025% (*w*/*v*) ammonium persulfate (APS); (ii) light gel solution: 357 mM Bis-Tris (pH 6.8), 8% (*w*/*v*) acrylamide/bis-acrylamide (37.5:1), 60 µM Phos-tag^TM^ (FUJIFILM Wako Pure Chemical Corporation, Japan), 0.01% (*w*/*v*) Coomassie Brilliant Blue G-250, 0.05% (*v*/*v*) TEMED, 0.05% (*w*/*v*) APS. Three volumes of heavy solution were poured between the gel plates, followed by one volume of light solution. After polymerization, the stacking gel containing 357 mM Bis-Tris (pH 6.8), 4% (*w*/*v*) acrylamide/bis-acrylamide (37.5:1), 0.1% (*v*/*v*) TEMED, and 0.05% (*w*/*v*) APS was prepared.

Thylakoid samples were incubated for 5 min at 70 °C in loading buffer containing 244 mM Tris HCl (pH 8.5), 10% (*w*/*v*) glycerol, 2% (*w*/*v*) lithium dodecyl sulfate, 0.33 mM Coomassie Brilliant Blue G-250, 100 mM dithiothreitol (DTT). One µg of Chl was loaded per well. Electrophoresis was performed with freshly prepared running buffer (61mM Tris, 50 mM MOPS, 0.1% (*w*/*v*) SDS, and 5 mM sodium bisulfite) at 55V for 4.5 h. After separation, proteins were transferred onto a PVDF membrane in a transfer buffer (25 mM bicine, 25 mM Bis-Tris, 1 mM EDTA, 10% (*v*/*v*) methanol, 5 mM sodium bisulfite; pH 7.2) at 150 mA constant current at 4 °C for 16.5 h. The membranes were blocked in Tris-buffered saline (TBS) buffer containing 3% (*w*/*v*) bovine serum albumin, 0.1% (*v*/*v*) Triton X-100 for 40 min with agitation, then dephosphorylated by incubation in a blocking solution supplemented with 400 U mL^−1^ of λ protein phosphatase (New England BioLabs, Ipswich, MA, USA, catalog number P0753L), 2 mM DTT, 2 mM MnCl_2_ for 4 h in room temperature. The membranes were washed with TBS before incubation with a primary antibody. Further steps of immunodetection were as described above.

### 4.5. Low-Temperature Fluorescence Measurements

Steady-state fluorescence emission spectra of chlorophyll at 77 K were recorded using a modified Shimadzu RF-5301PC spectrofluorometer as described previously [29]. Thylakoid crude fractions diluted to chlorophyll concentration of 10 µg mL^−1^ were placed in a polytetrafluoroethylene cuvette and submerged in liquid nitrogen. The excitation wavelength was set at 440 nm, excitation and emission slits at 5 nm, and scans were taken in the range of 600 to 800 nm (1 nm interval) through the LP600 emission filter. Mathematical deconvolution of the spectra was performed using the GRAMS/AI v. 9.2 (Thermo Fisher Scientific Inc., Waltham, MA, USA); spectra were brought to 0 at the 600 and 800 nm, normalized to a peak area value of 100, and decomposed into five Gaussian components [65]. The maxima of 685 and 695 nm components had 5 nm tolerance (680–685 and 690–695 nm), while the 700, 720, and 735 nm components’ maxima were fixed. For the best quality of Gaussian components fitting, the 680 and 685 nm bands were fitted with a single Gauss curve.

### 4.6. Chlorophyll a Fluorescence Measurements

Chlorophyll *a* fluorescence was recorded using the Maxi version of the Imaging-PAM chlorophyll fluorescence system (Heinz Walz, Effeltrich, Germany). Rosettes were dark-adapted for 25 min before measurements. Minimal (F_0_) fluorescence was measured using weak blue modulating light of 0.5 μmol photons m^−2^ s^−1^, whereas maximal (F_M_) fluorescence was measured using 0.84 s saturation blue light pulse of 2700 μmol photons m^−2^ s^−1^. After 60 s of dark relaxation, the blue actinic light of 130 μmol photons m^−2^ s^−1^ was on, and saturation pulses were applied at 20 s time intervals. After 200 s, actinic light was switched off, and additional saturation pulses for approximately 15 min were used. At all saturation pulses, the maximum (F_M_′) fluorescence values were measured, and the minimum (F_0′_) fluorescence was calculated as F_0_′ = F_0_/(F_V_/F_M_ + F_0_/F_M_′). The recorded data were analyzed using ImagingWinGigE software, and the photosynthetic parameters F_V_/F_M_ = (F_M_ − F_0_)/F_M_, NPQ = (F_M_/F_M_′) – 1, and qL = (F_M_′ − F)/(F_M_′ − F_0_′) × F_0_′/F were calculated. The qE and qI components of NPQ were calculated according to [37].

### 4.7. Thiobarbituric Acid-Reactive Substances (TBARS) Assay

Approximately 50 mg of fresh leaf tissue was frozen in liquid nitrogen and stored at −80 °C for a maximum of 2 weeks. Tissue was homogenized in 80% (*v*/*v*) ethanol, 0.01% (*w*/*v*) butylated hydroxytoluene using steel beads and SpeedMill homogenizer (Analytik Jena, Jena, Germany), followed by centrifugation at 12,000× *g* for 5 min at room temperature. Equal volumes of supernatant were mixed with 0.65% (*w*/*v*) thiobarbituric acid in 20% (*w*/*v*) trichloroacetic acid (TCA) or only with 20% (*w*/*v*) TCA, followed by incubation at 95 °C for 20 min. Next, samples were cooled on ice for 10 min and centrifuged at 7000× *g* for 15 min at 4 °C. The concentration of TBARS in terms of MDA content was calculated according to [66] using absorbance measured at 600 nm, 532 nm, and 440 nm with the help of a Cary50 Bio spectrophotometer.

### 4.8. Statistical Analysis

The statistical significance was verified by one-way ANOVA with post hoc Tukey test at *p* = 0.05. The number of repetitions of specific experiments is given in the figure legends.

## Data Availability

Not applicable.

## Supplementary Materials

The following supporting information can be downloaded at: https://www.mdpi.com/article/10.3390/ijms23094531/s1.
