# Supplementary material for: STN7 Kinase Is Essential for Arabidopsis thaliana Fitness under Prolonged Darkness but Not under Dark-Chilling Conditions
Source: Int J Mol Sci. 2022 Apr 20;23(9):4531. doi: 10.3390/ijms23094531 (PMC9100030; doi:10.3390/ijms23094531)
Supplement: Supplementary file 1 [file ijms-23-04531-s001.zip › ijms-1639335-supplementary.pdf]

Supplementary material for article

STN7 Kinase is Essential for *Arabidopsis thaliana* Fitness under Prolonged Darkness but Not under Dark-Chilling Conditions

Anna Węgrzyn, Małgorzata Krysiak, Anna Kulik, Katarzyna B. Gieczewska, Radosław Mazur*

*Correspondence: rmazur@uw.edu.pl , Tel: +48-225543213

Table S1. Densitometric analysis of gel presented in Figure 1

	Pea	Bean	Col-0	*chs2-1*
Intensity of phosphorylated LHCII band[1]				
0h	125409894.86	99479320.12	85650175.96	52009750.52
72h	135974054.32	170718384.78	73229857.81	65487944.98
Intensity of LHCII band[2]				
0h	230859183.67	210461501.97	156952598.43	153998304.59
72h	211772249.86	227501408.43	187553159.98	159886558.98
LHCII phosphorylation [%][3]				
0h	54.32	47.27	54.57	33.77
72h	64.21	75.04	39.04	40.96

[1]Intensity of phosphorylated LHCII (P-LHCII) bands measured on a gel stained with ProQ Diamond (A)
[2]Intensity of LHCII bands measured on a gel stained with Sypro RUBY (B)
[3]LHCII phosphorylation calculated as follows: (A) / (B) x 100.

Table S2. Densitometric analysis of blots presented in Figure 2

	Col-0				*chs2-1*				*stn7-1*				*tap38-2*			
	Raw data		Normalized to 0h		Raw data		Normalized to 0h		Raw data		Normalized to 0h		Raw data		Normalized to 0h	
	Dark-chilling	Dark	Dark-chilling	Dark	Dark-chilling	Dark	Dark-chilling	Dark	Dark-chilling	Dark	Dark-chilling	Dark	Dark-chilling	Dark	Dark-chilling	Dark
Intensity of phosphorylated CP43 band[1]																
0h	2379997	1648049	1.00	1.00	1438586	1042759	1.00	1.00	3366942	1940590	1.00	1.00	2894675	3817023	1.00	1.00
3h	3008365	1790541	1.26	1.09	1489758	1474347	1.04	1.41	3459382	4081471	1.03	2.10	2829135	3605117	0.98	0.94
6h	2856697	1843745	1.20	1.12	1409794	1955171	0.98	1.87	3556670	3334255	1.06	1.72	2783327	2689750	0.96	0.70
9h	2701617	1653797	1.14	1.00	1638874	1853603	1.14	1.78	2654818	3468555	0.79	1.79	2476507	2726354	0.86	0.71
12h	3606219	2065741	1.52	1.25	2098426	1544643	1.46	1.48	3273370	3999663	0.97	2.06	2563571	2740982	0.89	0.72
24h	2485019	1667621	1.04	1.01	1377614	1679527	0.96	1.61	3353906	2644291	1.00	1.36	2375387	3106732	0.82	0.81
48h	3100179	1501705	1.30	0.91	1568590	1649399	1.09	1.58	2948286	2310935	0.88	1.19	2487387	3040962	0.86	0.80
72h	2962339	1425337	1.24	0.86	2093490	1769983	1.46	1.70	2906790	2510383	0.86	1.29	3204386	2993216	1.11	0.78
Intensity of phosphorylated D1/D2 band[1]																
0h	2465485	1967510	1.00	1.00	1240550	646664	1.00	1.00	3964332	2051185	1.00	1.00	3722860	4315753	1.00	1.00
3h	2527387	2186470	1.03	1.11	1460686	1276184	1.18	1.97	4098087	4584421	1.03	2.24	3483876	4296831	0.94	1.00
6h	2788571	2419350	1.13	1.23	1001958	1353200	0.81	2.09	4011503	4328705	1.01	2.11	3476316	3456348	0.93	0.80
9h	2891691	2317782	1.17	1.18	1269722	1498448	1.02	2.32	2699007	4486117	0.68	2.19	3431620	4236504	0.92	0.98
12h	3747943	2427398	1.52	1.23	1662382	1522156	1.34	2.35	3118479	4485769	0.79	2.19	3402028	3963860	0.91	0.92
24h	2132739	2603858	0.87	1.32	1341234	1484116	1.08	2.30	2954807	3577701	0.75	1.74	3033920	4829775	0.81	1.12
48h	2321503	2724114	0.94	1.38	1556638	1374176	1.25	2.13	2778311	2819261	0.70	1.37	2780820	5005040	0.75	1.16
72h	2432879	2423974	0.99	1.23	2254327	1790723	1.82	2.77	2733839	3187205	0.69	1.55	2702768	4378334	0.73	1.01
Intensity of phosphorylated LHCII band[1]																
0h	1021389	931602	1.00	1.00	749019	391788	1.00	1.00	–	–	–	–	1544377	1226032	1.00	1.00
3h	972865	844786	0.95	0.91	1047411	900204	1.40	2.30	–	–	–	–	1309925	1324962	0.85	1.08
6h	993249	1268218	0.97	1.36	649711	1271384	0.87	3.25	–	–	–	–	1701189	1059135	1.10	0.86
9h	788337	902894	0.77	0.97	842407	886356	1.12	2.26	–	–	–	–	1330469	936708	0.86	0.76
12h	1962177	519390	1.92	0.56	1176055	806320	1.57	2.06	–	–	–	–	1120317	622673	0.73	0.51
24h	735361	1208838	0.72	1.30	824551	1536160	1.10	3.92	–	–	–	–	747969	1144700	0.48	0.93
48h	703813	1583378	0.69	1.70	872659	1427524	1.17	3.64	–	–	–	–	514961	1093728	0.33	0.89
72h	1016837	2056886	1.00	2.21	1113027	2423272	1.49	6.19	–	–	–	–	452977	2542955	0.29	2.07
Intensity of Lhcb1 band[2]																
0h	1882955	1244634	1.00	1.00	2682156	264555	1.00	1.00	1906739	1398460	1.00	1.00	2292140	610002	1.00	1.00
3h	1848806	1190178	0.98	0.96	3185646	245187	1.19	0.93	2038491	1740085	1.07	1.24	3061416	670590	1.34	1.10
6h	2029536	933682	1.08	0.75	3080412	368739	1.15	1.39	2260483	2173260	1.19	1.55	2648216	554854	1.16	0.91
9h	2103682	1032288	1.12	0.83	3144046	405399	1.17	1.53	1924259	2352619	1.01	1.68	2610936	645486	1.14	1.06
12h	2288433	1224826	1.22	0.98	3114121	477204	1.16	1.8	2216245	2394667	1.16	1.71	3100820	565134	1.35	0.93
24h	1755215	894865	0.93	0.72	3677336	414563	1.37	1.57	2012073	2156666	1.06	1.54	3682452	473748	1.61	0.78
48h	1652403	769874	0.88	0.62	3229701	340040	1.2	1.29	1705713	2238093	0.89	1.60	3466496	610124	1.51	1.00
72h	1765181	962289	0.94	0.77	3537168	327639	1.32	1.24	1392261	2066354	0.73	1.48	4771211	741828	2.08	1.22

[1] bands intensities of phosphorylated forms of CP43, D1/D2, and LHCII proteins were measured on an anti-phospho-threonine blot using ImageLab software

[2] bands intensities of Lhcb1 protein were measured on an anti-Lhcb1 blot using ImageLab software.

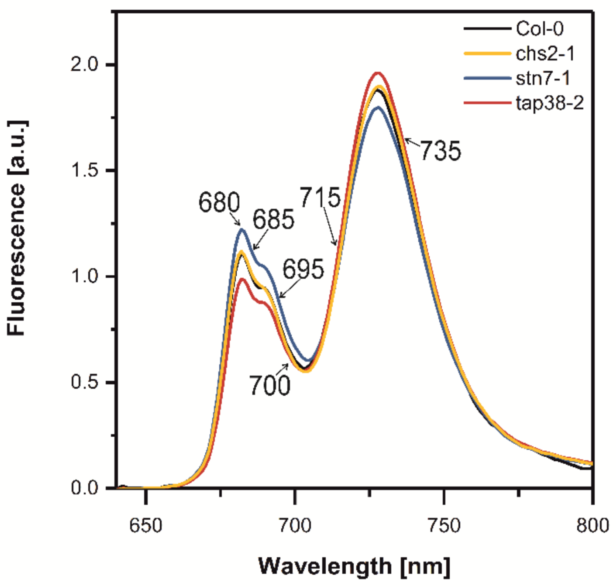

Figure S1. Low temperature chlorophyll *a* fluorescence emission spectra of thylakoid membranes. Crude thylakoid fractions were isolated from wild type Col-0, *chs2-1*, *stn7-1,* and *tap38-2* lines of Arabidopsis plants from control conditions (time-point 0h). Emission maxima of the main components of the spectrum are indicated by arrows (details in the main text).

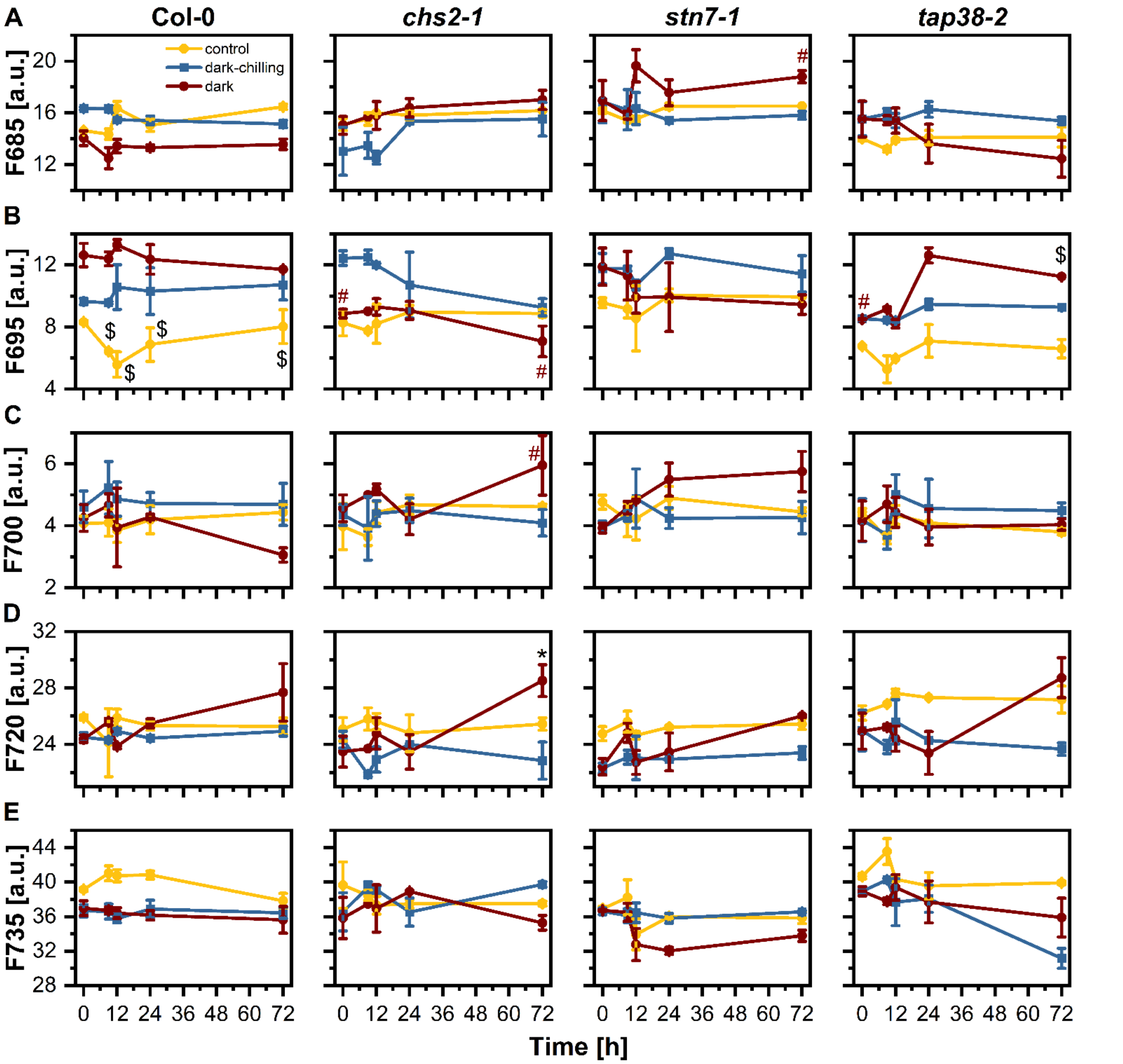

Figure S2. Changes of five main fluorescence bands contributions to low-temperature chlorophyll *a* fluorescence emission spectra of thylakoid membranes isolated from wild type Col-0, *chs2-1*, *stn7-1*, and *tap38-2* lines of Arabidopsis plants from control, dark-chilling, and dark conditions. Fluorescence bands F685 (A), F695 (B), F700 (C), F720 (D), and F735 (E) were obtained by Gaussian deconvolution of the recorded spectra. Data are mean values ± SE from two independent experiments. Results marked with an (i) asterisk, (ii) dollar, and (iii) hash differ significantly at $p = 0.1$ (one-way ANOVA with post hoc Tukey test for time-points 0 h and 72 h): (i) dark-chilling vs. dark conditions (ii) dark vs. control conditions, (iii) mutant vs. wild type, respectively. The color code for statistics is the same as for the data.

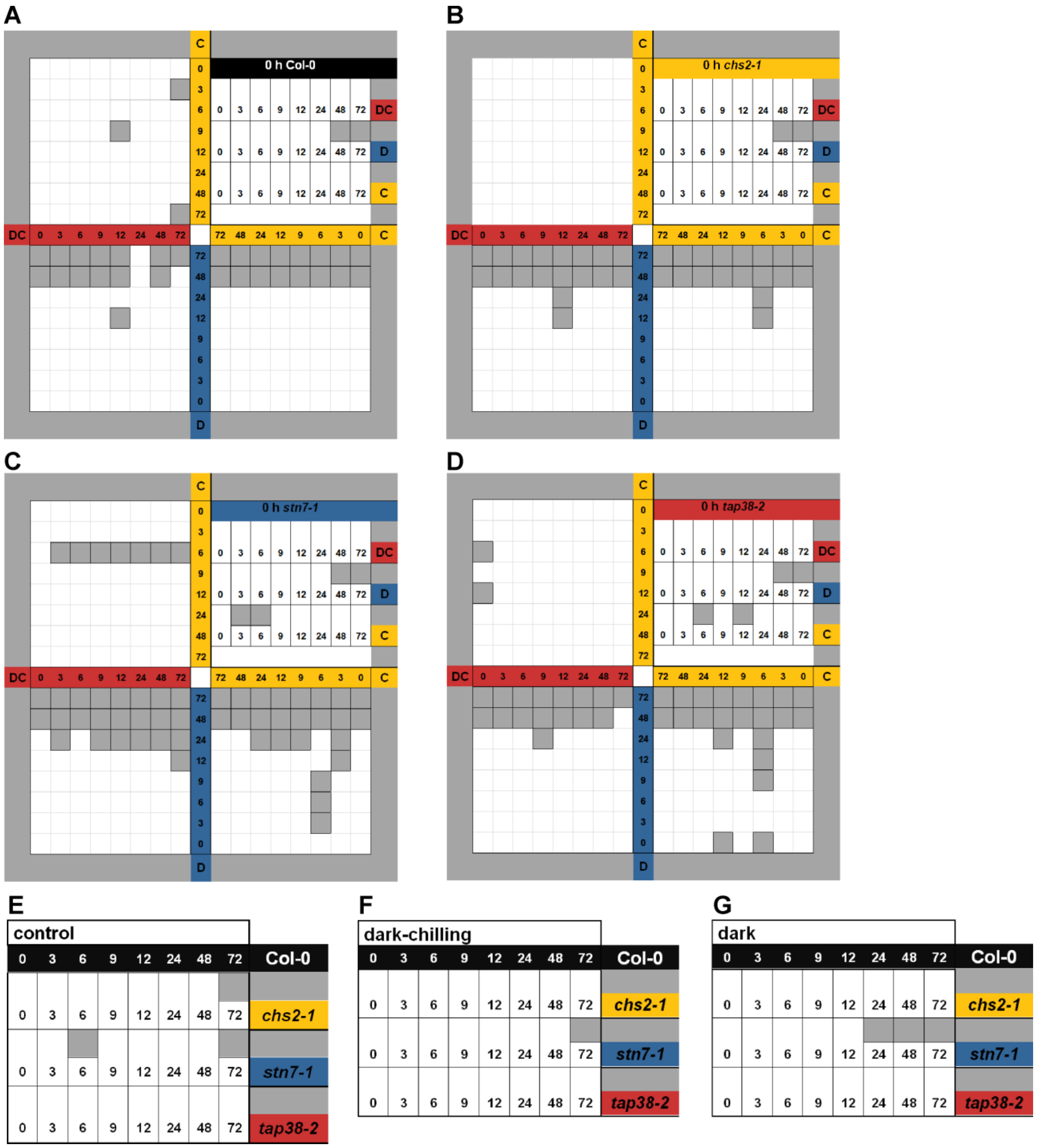

Figure S3. The statistical significance (one-way ANOVA with post hoc Tukey test at $p = 0.05$) of the changes in F_V/F_M is presented in Figure 6 in the main text. A-D, graphs show the significance of F_V/F_M differences, marked as grey shade, in Col-0 (A), *chs2-1* (B), *stn7-1* (C), and *tap38-2* (D) in control vs. dark-chilling (top left), control vs. dark (bottom right) and dark-chilling vs. dark (bottom left) conditions. The top right panel of each graph (A-D) shows significant differences between time-point and 0 h, separately for all analyzed lines. E-G, graphs show significant differences mutant vs. Col-0 in control (F), dark-chilling (F), and dark (G) conditions.

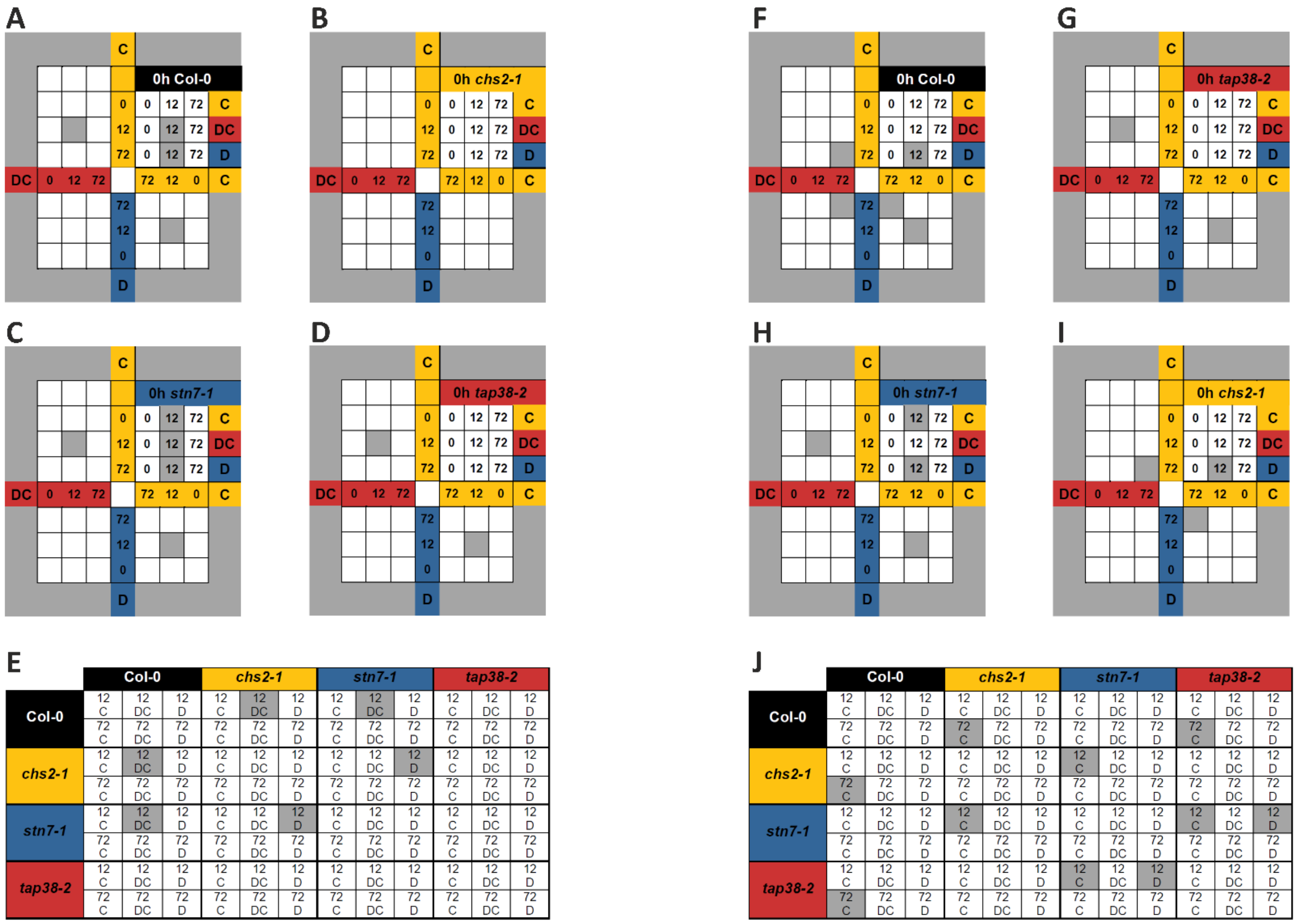

Figure S4. The statistical significance (one-way ANOVA with post hoc Tukey test at $p = 0.05$) of the changes in NPQ is presented in Figure 7 in the main text. Graphs show the significance of steady-state NPQ (A-E) and maximal NPQ (F-J) differences, marked as grey shade, in Col-0 (A, F), *chs2-1* (B, G), *stn7-1* (C, H), and *tap38-2* (D, I) in control vs. dark-chilling (top left), control vs. dark (bottom right) and dark-chilling vs. dark (bottom left) conditions. The top right panel of each graph (A-D and F-I) shows significant differences between time-point and 0 h, separately for all analyzed lines. E and J graphs show significant differences in mutant vs. Col-0 in all experimental conditions.

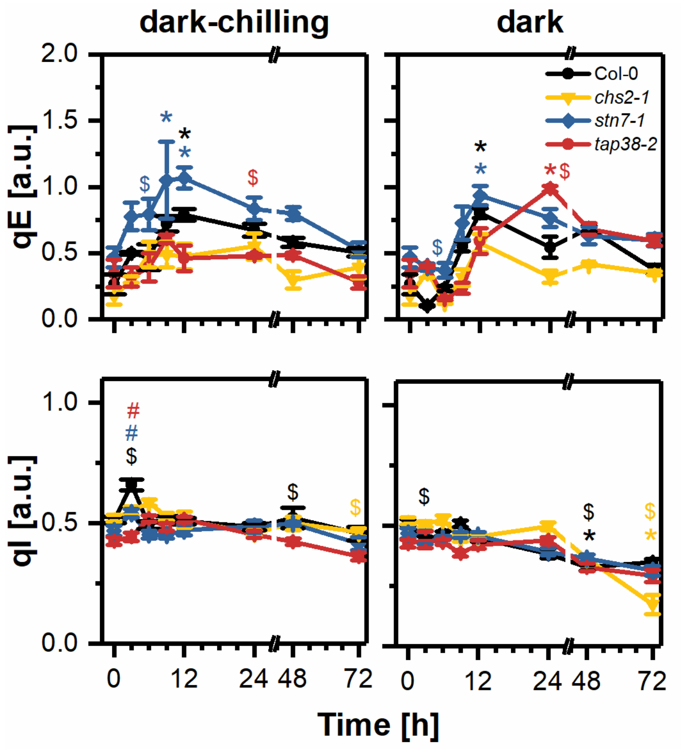

Figure S5. Changes in qE and qI components of NPQ. qE and qI were calculated for wild type Col-0, *chs2-1*, *stn7-1*, and *tap38-2* lines of Arabidopsis plants from dark-chilling and dark conditions. Data are mean values ± SE from three independent experiments. Results marked with an (i) asterisk, (ii) hash, and (iii) dollar differ significantly at $p = 0.05$ (one-way ANOVA with post hoc Tukey test): (i) time-point vs. 0 h within the plant line and experimental conditions, (ii) mutant vs. Col-0 within the time-point and experimental conditions, (iii) dark-chilling vs. dark conditions within plant line and time-point, respectively. The color code for statistics is the same as for the data.

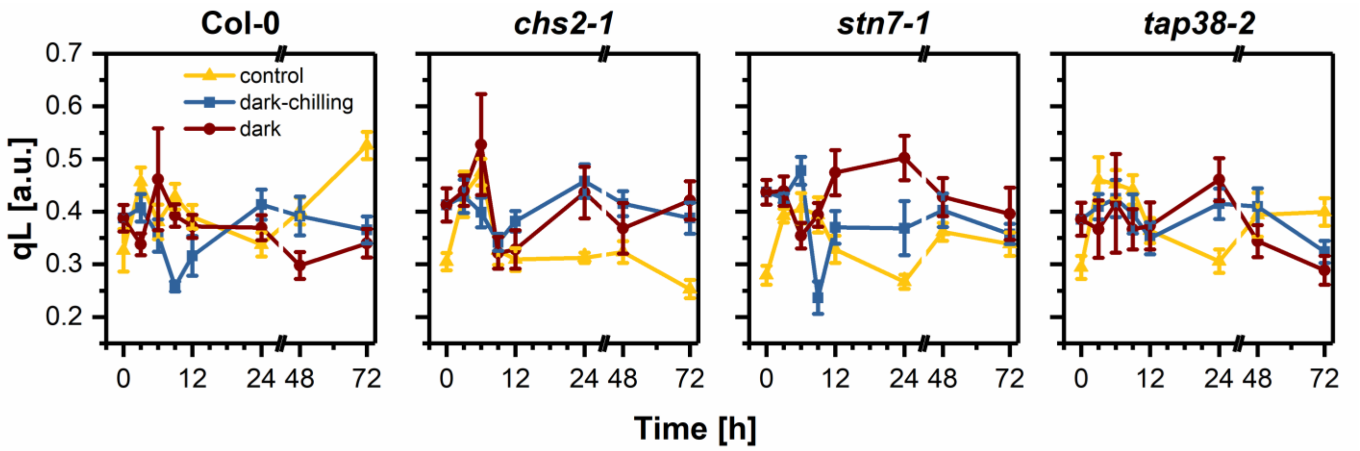

Figure S6. Changes in qL (parameter representing the fraction of open PSII reaction centers) were calculated for light-adapted plants of Col-0, *chs2-1*, *stn7-1*, and *tap38-2* lines of Arabidopsis plants from control, dark-chilling, and dark conditions. Data are mean values ± SE from three independent experiments. Significant differences in one-way ANOVA with post hoc Tukey test at $p = 0.05$ were not reported.

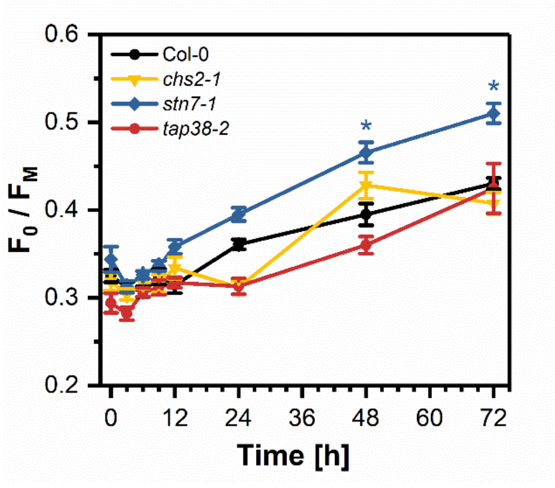

Figure S7. Changes in F_0/F_M values in Col-0, *chs2-1*, *stn7-1*, and *tap38-2* lines of Arabidopsis plants from dark conditions. Data are mean values ± SE from three independent experiments. Results (mutant vs. Col-0 within time-point) marked with an asterisk differ significantly at $p = 0.05$ (one-way ANOVA with post hoc Tukey test) mutant vs. Col-0. The color code for statistics is the same as for the data.
